# Monitoring storm evolution using a high-density seismic network

**DOI:** 10.1038/s41598-023-28902-8

**Published:** 2023-02-01

**Authors:** J. Diaz, M. Ruiz, M. Udina, F. Polls, D. Martí, J. Bech

**Affiliations:** 1 Geosciences Barcelona - CSIC, Barcelona, Spain; 2 Department of Applied Physics – Meteorology, University of Barcelona, Barcelona, Spain; 3 Water Research Institute, University of Barcelona, Barcelona, Spain

**Keywords:** Seismology, Atmospheric dynamics, Natural hazards

## Abstract

Data acquired by a dense seismic network deployed in the Cerdanya basin (Eastern Pyrenees) is used to track the temporal and spatial evolution of meteorological events such as rainfall episodes or thunderstorms. Comparing seismic and meteorological data, we show that for frequencies above 40 Hz, the dominant source of seismic noise is rainfall and hence the amplitude of the seismic data can be used as a proxy of rainfall. The interstation distance of 1.5 km provides an unprecedented spatial resolution of the evolution of rainfall episodes along the basin. Two specific episodes, one dominated by stratiform rain and the second one dominated by convective rain, are analyzed in detail, using high resolution disdrometer data from a meteorological site near one of the seismic instruments. Seismic amplitude variations follow a similar evolution to radar reflectivity values, but in some stratiform precipitation cases, it differs from the radar-derived precipitation estimates in this region of abrupt topography, where radar may suffer antenna beam blockage. Hence, we demonstrate the added value of seismic data to complement other sources of information such as rain-gauge or weather radar observations to describe the evolution of ground-level rainfall fields at high spatial and temporal resolution. The seismic power and the rainfall intensity have an exponential relationship and the periods with larger seismic power are coincident. The time intervals with rain drops diameters exceeding 3.5 mm do not result in increased seismic amplitudes, suggesting that there is a threshold value from which seismic data are no longer proportional to the size of the drops. Thunderstorms can be identified by the recording of the sonic waves generated by thunders, with. Single thunders detected to distances of a few tens of kilometers﻿.﻿ As the propagation of these acoustic waves is expected to be strongly affected by parameters as air humidity, temperature variations or wind, the seismic data could provide an excellent tool to investigate atmospheric properties variations during thunderstorms.

## Introduction

Background seismic vibrations in the absence of earthquakes, often referred as “ambient noise” are detected on seismograms recorded by seismic stations whatever their locations, on continents, on islands or on the ocean bottom. Within the 0.1–1 Hz frequency band, the source of ambient noise is related to ocean waves, while at higher frequencies, large amplitude noise is mostly related to weather or anthropogenic activities^1^. In the past 15 years, seismic ambient noise has revealed as a useful tool for monitoring and imaging the subsurface crustal structure^2^, as it is a non-invasive and low environment impact technique that do not need the use of active sources as explosives or vibration trucks.

The origin of so-called “environmental seismology”^3^ can be found in the investigation of the microseismic peak, the frequency range with higher energy, which was soon related to the oceanic activity^4^. In the last decade environmental seismology has emerged as an active research field, based on the use of the vibrations generated by different processes, and recorded at seismic stations to monitor to some detail their activity. This approach has been used to study a large variety of processes, many of them related to hydrology and meteorology sciences, as Antarctica ice cover^5^, glaciers^6^, river floods^7^, mountain snowmelt^8^, groundwater variations^9^, rain^10^, wind^11^ and thunders^12^. This range of phenomena can be extended to topics related within astronomy and astrophysics, as the monitoring of auroras, electromagnetic storms or bolides entering the atmosphere^12–14^ and also to the biological sciences, using seismic data to detect marine mammal vocalizations^15^ or to investigate the use of vibrations as a communication tool by the elephants^16,17^.

In this contribution, we show how seismic data acquired in dense networks can be used to monitor the evolution of meteorological events, including rainfall and thunderstorm, with an unprecedented detail. We will use data acquired in the Cerdanya basin (eastern Pyrenees) between April and late June 2021 in the framework of the SANIMS project (Spanish M. of Science, Innovation and Universities, Ref.: RTI2018-095,594-B-I00), that focused on the application and development of methods based on ambient noise seismic data to image and monitor natural and human-altered environments. The motivation to use this dataset as a tool to investigate meteorological events comes from the fact that during the quality control procedure of the data, we noticed a time correspondence between intervals of increased noise and the rainfall episodes experienced by the field teams.

The Cerdanya basin is a relatively small Neogene basin located in the eastern part of the Pyrenean Axial Zone, extending about 35 km along the NE-SW / ENE-WSW direction with a maximum width of 5–7 km and an approximate area of around 250 km^2^. The basin is crossed by the Segre River, one of the main tributaries of the Ebro River and its mean altitude is of 1100 m, with surrounding mountains reaching 2500–2900 m. It is limited to the East by the Alp-Têt fault, a major accident in the eastern Pyrenees formed by NE-SW right-stepping en-echelon faults and E-W oriented faults that play a major role in the recent tectonic evolution of the zone. The maximum thickness of the basin, located in its SW zone, has been estimated to 650 m from 2D seismic and magnetotelluric profiles^18^.

Due to surrounding orography and geographical location of the basin, wind circulation is driven by slope and along-valley winds^19^ under weak synoptic winds. Extreme minimum absolute temperatures occur during winter, leading to strong cold-air pools^20^. Precipitation events occur both in warm and cold seasons, some of them associated with meridional synoptic flows impinging perpendicularly to the mountain ranges^21–23^.

## Datasets

### Seismic dataset

**Figure 1:**
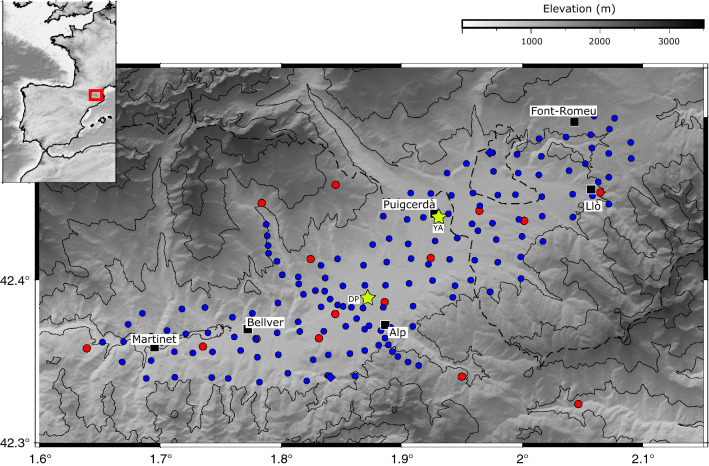
Cerdanya basin topographic map showing the broad-band stations (red dots) and the high-density seismic network (blue dots). Green stars show the location of the Puigcerdà (YA code) and Das (DP code) meteorological stations. Black boxes and the corresponding labels show the locations of towns. The topographic database was retrieved from CIAT-CSI SRTM (http://srtm.csi.cgiar.org)^24^.

During the SANIMS project, the Cerdanya Basin has been explored using two different seismic networks, each designed with specific objectives. First, a network of broad-band seismic stations, including 10 sites in the Cerdanya zone and 14 additional sites distributed around, was operated between September 2019 and November 2020 (red dots in Fig. 1). The deployment includes stations distributed along the axis of the basin, with an interstation distance close to 4 km.

Later on, a denser deployment using up to 140 high frequency seismic sensors was operated between April and June 2021, covering an area of 300 km^2^ in the Cerdanya basin with a station spacing of about 1.5 km. The data is acquired continuously with a sampling frequency of 250 samples per second. The sensors used can record accurately signals with frequencies above 1 Hz. The spacing between stations allow for the detection of variations in seismic amplitude with an excellent resolution and will hence provide the backbone of the results discussed here.

**Figure 2:**
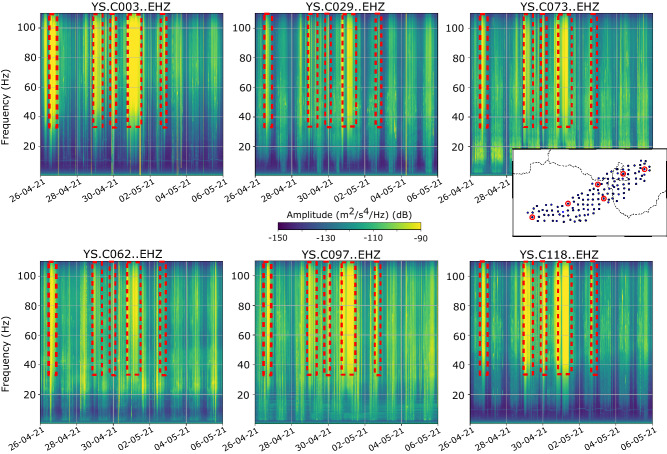
Spectrograms corresponding to the 26/4–6/5/2021 period for representative stations distributed along the network. Yellowish colors correspond to large energy levels. The time intervals with higher energy at frequencies above 40 Hz (red dashed boxes) can be identified at each site.

Although data from the three components (vertical, north/south and east/west) were acquired in both deployments, this study focuses on analysis of the vertical components. As part of the quality checking procedure, the spectrograms of the seismic stations have been systematically calculated, using window lengths of 30 min, with a 50% of overlapping. Figure 2 shows the spectrograms for a 10-day period at six representative sites covering different zones of the basin. It is easy to observe that during specific time periods, large values of energy at frequencies above 40 Hz are observed simultaneously at all the stations. At the stations located in quiet areas (e.g. C003, C118 in Fig. 2) these episodes of increased energy can be identified down to frequencies around 10 Hz. However, at stations located in nosier settings (e.g., C073 in Fig. 2), the effect of anthropogenic sources clearly dominates at frequencies below 30–40 Hz, as shown by the 24 h period of the energy variations. Meng et al.^25^ have shown that seismic signals generated by cars cannot be detected for frequencies above 30 Hz at distances of only few hundred of meters. Therefore, a plausible hypothesis is that these periods of large amplitude observed at multiple stations relate to meteorological phenomena, such as wind bursts or rainfall episodes. To check this hypothesis, we have filtered the seismic data to keep only frequencies above 40 Hz and we have gathered the meteorological data available in the area.

### Meteorological dataset

The meteorological station network managed by the Meteorological Service of Catalonia (SMC)^26,27^ includes two automatic stations near Puigcerdà and Das, both located in the central part of the basin and are separated by about 8 km (Fig. 1). Regarding rainfall, the available data refers to accumulated precipitation in 30-min intervals. Wind speeds are measured every 30 min at 10 m above ground level. The data during the period of interest is presented in Suppl. Figure 1.

We have also used 2-km height Constant Altitude Plan Position Indicator (CAPPI) radar reflectivity observations obtained with the SMC Doppler radar network^28^. SMC CAPPI radar data are available with a spatial resolution of 4 square kilometers and are updated every 6 min. Radar reflectivity provided by weather radars is used traditionally to derive rainfall rate at ground level. However, this conversion is not straightforward, as several assumptions are made, so uncertainty is usually high, particularly in complex terrain areas. For example, due to the earth curvature and atmospheric refraction, radar reflectivity is observed at an increasing height above the radar level as longer ranges are considered. Therefore, a correction of the observed reflectivity aloft compared to the estimated value at ground level must be performed before converting reflectivity to precipitation rate. Weather radar partial beam blockage may affect the quality of radar-derived precipitation estimates^29,30^, particularly for low-topped precipitation that is typically caused by stratiform clouds. Additionally, intense precipitation echoes with high rainfall rates may also produce attenuation of C-band radar observations along the radar line of sight^31^. We have processed the reflectivity data to classify echo type into convective, stratiform and mixed precipitation regimes according to Powell et al.^32^. Observations from the SMC Lightning detection network^33^ have also been used to identify the occurrence (position and timing) of lightning flashes resulting in sonic shock waves (thunder).

In addition, we have used the measurements from a Particle Size Velocity (Parsivel) optical disdrometer^34^ managed by the University of Barcelona in Das, near the center of the seismic network. This instrumentation, manufactured by OTT^35^, provides detailed information, with 1 min temporal resolution, on the rain drop size spectra from 0.25 mm to 6.00 mm. Disdrometer data provide a much richer information than traditional rain gauge records, allowing a better interpretation of the seismic data.

### Overview of selected precipitation events

During the investigated period, rainfall is mostly concentrated in three rainfall events: 25 April-1 May, 28 May-6 June and 12–21 June, with accumulated precipitation values reaching 247.6 mm at Puigcerdà and just 174.1 mm in Das. While during the two first episodes the accumulations registered at both locations are similar, the mid-June episode results in large differences, with the Puigcerdà recording three times more precipitation than Das.

The late April episode has a rather homogeneous rainfall time series, with 30 min accumulation not exceeding 3 mm but without large gaps without precipitation, in particular between the 28 April and 01 May. The other two episodes are more irregular, with shorter but more intense precipitation intervals. The larger rainfall episode is recorded 17 May, with accumulated 30-min precipitation values reaching 25 mm in the Puigcerdà rain gauge, that is, 5 times larger than for the late April episode. Regarding wind speeds, both sites show a strong day/night variation, with maximum values reaching 7 m/s at Das and 10 m/s at Puigcerdà between the 10 and 20 May, and not exceeding 5 m/s during the rest of the investigated period. The time variation of the wind speed is consistent between both sites, although the values are systematically greater at Das.

In the following we will focus on the late April and mid-June episodes, selecting two 7-day long intervals, from 25 April to 01 May and from 14 to 20 June. To compare seismic and meteorological data, the seismic signals have been high-pass filtered above 40 Hz, downsampled and represented by their envelope function to hence display their amplitude as positive values. As all the instruments were identical, we prefer do not remove the instrumental response to avoid eventual numerical artifacts and keep their amplitude units in digital counts.

**Figure 3:**
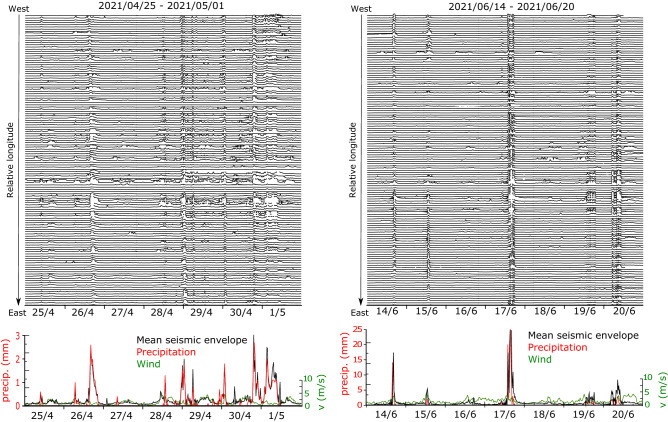
Top panels: Envelopes of the seismic signals during the late April (left) and mid-June rainfall episodes (right), downsampled to one sample every 20 min. Traces are ordered following longitude, with westward sites on top. All traces are displayed using the same amplitude scale, expressed in digital counts. Lower panels: precipitation (red line) and wind speed (green line) at Puigcerdà and the mean seismic envelope calculated using the five sites closer to the meteorological station (black line). Vertical scales have been adjusted to highlight the correlation between seismic and meteorological data.

Figure 3 shows the seismic data for the late April and mid-June episodes. Each line in the upper panels corresponds to a seismic station, ordered accordingly to their longitude, with the westernmost sites on top of the figure. All the stations detect the same episodes of large amplitude, but with significant differences in the relative amplitude and the time arrival of the maximum peaks. The bottom panels show the meteorological data (precipitation in red, wind speed in green) for the Puigcerdà station, plotted together with the seismic envelopes, calculated as the mean envelope of the five seismic sites closest to the meteorological station. Larger seismic amplitudes correlate well with the precipitation peaks. Although the correspondence between precipitation and seismic amplitude values is not fully consistent over time, rainfall is ostensibly responsible of the largest part of the seismic motion in the selected frequency band.

Several studies have also argued that near-ground wind contributes significantly to seismic amplitudes, both in low and high frequency bands^36,37^, although this contribution depends highly on each specific site, being usually larger in forest areas or zones with tall installations (e.g. antennas, and wind turbines…). A correlation between wind and seismic amplitude is present occasionally in our data, as can be seen in the left panel of Fig. 3. Relatively small increases in wind speed that appear near the 25 April, the 27 April, or the 29 April tick marks are reproduced by the seismic envelope variations. However, the contribution of the wind-related shaking to the total seismic amplitude is very limited. During the studied time interval, mean wind velocities were around 5–7 m/s, with maximum values not exceeding 10 m/s. In case of stronger winds, it is probable that their contribution to ground shaking would be more relevant. Therefore, we conclude that the major source of seismic vibrations for frequencies above 40 Hz is rainfall, and we propose that these seismic data can be considered as a proxy for rainfall and hence used to track the spatial and time evolution of rainfall episodes.

## Spatial and time evolution of rainfall episodes

In order to study the time and spatial evolution of rainfall episodes in detail, we have used the envelopes shown in Fig. 3 to evaluate the mean value of the seismic amplitudes for each station of our network, at time intervals ranging between 5 and 30 min. We have represented these values in a map, hence creating a series of snapshots illustrating the evolution of the episode. In order to smooth the results, a grid has been interpolated at each selected time, using the nearest neighbor algorithm implementation included in the GMT software package^38^. We have used a search radius of 3.5 km and required that 5 of 6 sectors are filled to perform an average. This parametrization avoids the extension of the grid to unconstrained zones and assures a general consistency of the results. To identify possible artifacts related to grid interpolation, the maps also label the values at each station with circles colored using the same palette. Assuming that seismic noise above 40 Hz is a proxy of rainfall, the resulting maps allow us to track with unprecedented detail the rainfall spatial distribution over the area.

As shown in recent publications using seismic data to monitor the COVID19 lockdown measures, human activity can contribute to seismic vibrations and perturb the use of seismic data as a proxy for rainfall. We have checked that the noise variation in the selected frequency band does not reproduce the geometry of main roads or activity areas, as would be expected if traffic and industrial activities were the main source of vibrations. The high-density network allows us to identify a limited number of problematic sites, which display very often higher-than-average amplitudes. Based on both their waveforms and spectra, we have identified stations for which site amplification effects or local anthropogenic sources dominates the high-frequency seismic signals. These problematic stations (5.7% of the total) include sites close to main roads (C064) or industrial parks (C072), sites affected by water pumps used in golf courses (C057) or spa installations (C119), and a station close to local road with ongoing civil works. We have excluded these stations in the final maps and animations.

### April episode

This episode shows a large peak the 26 April, followed by 2 days of precipitation quiescence, a period with limited but rather constant rainfall, and a final episode extending between late 30 April and midday 1st May. The two meteorological stations record similar total rainfall amounts during the episode, and measure 63.7 mm at Puigcerdà and 63.5 mm at Das. The seismic amplitudes mimic this general pattern and provide high resolution details on the temporal and spatial evolution of the storm (Fig. 3, left panel).

During the 26 April rainfall, seismic envelopes show that rain starts first to the west and progresses eastward. Envelopes reach peak amplitudes around 14:45 UTC at the westernmost station, reaches the Bellver de Cerdanya zone around 15:15, Puigcerdà around 16:00 and the stations located to the eastern limit of the network around 17:15. Since the distance between the network boundaries is about ~ 40 km, we can estimate that the peak of this storm traveled at an apparent speed of about 4.5 m/s (16 km/h), a value consistent with previous studies in the region^39^. The larger seismic energy values are observed in the stations located around Puigcerdà, where the meteorological data indicated rain accumulations of 13.5 mm during this 3 h-long episode. The rainfall events of the 28 and 29 April show a similar tendency, but with shorter time delays of less than 2 h. The rainfalls registered the 30 April- 1st May do not seem to move along the region.

**Figure 4:**
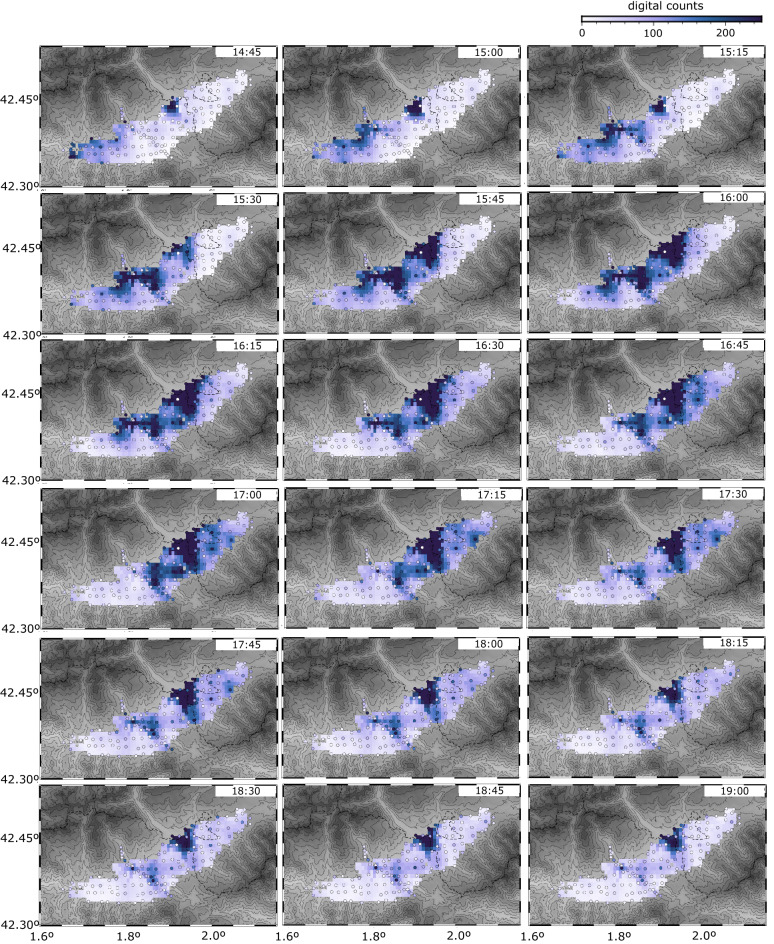
Snapshots of seismic noise maps during the 26 April rainfall episode. Each image shows the mean amplitude of the vertical seismic component for 15 min intervals for frequencies above 40 Hz. Dark colors represent large amplitude values and are interpreted to relate to rainfall. A grey color palette indicates topography (see Fig. 1).

The maps of the seismic amplitude values calculated in 15 min-long intervals have been used to build an animation displaying the spatial and temporal evolution of the episode, presented as Suppl. Material 1. Although the animation contains multiple points of interest, we will highlight the most intense rain event, which occurred on April 26 2021, approximately between 13:00 and 21:00 UTC, when 13.5 mm fell in Puigcerdà and 12.8 mm in Das. Figure 4 shows the time and spatial evolution of the central part of this event. At 14:45 UTC, large areas in central and western Cerdanya show increased seismic amplitude levels associated to rainfall (light blue colors), with strong signals to the west and near the Carol River valley, north of Puigcerdà, while there is no evidence of rain at the eastern part of the basin. At 15:15/15:30 UTC, the stations aligned with the Carol River and the Duran River valleys (both oriented NW–SE) begin to show large amplitudes (dark blue colors), then extend to the central part of the Cerdanya basin. After 16:30 UTC, while the amplitudes in this zone remain constant, seismic noise progressively disappears of the western part and shifts to the NE stations. This pattern persists until approximately 18:30–19:00, when rain intensity progressively decreases everywhere. By 20:00, most of the sites show low amplitudes, although the stations north of Puigcerdà continue to present high levels of noise. The persistence of noise in this specific area, not particularly affected by human activity, suggests that the NW oriented valleys provided a pathway for the progress of the more intense episodes affecting the northern side of the Pyrenees to the Cerdanya basin.

Supplementary Fig. 2 compares radar and seismic data for this episode. The rain type maps all show stratiform precipitation, with just a couple of short duration and very localized convective rain detections. The constant altitude plan position indicator (CAPPI) reflectivity maps at an equivalent height of 2 km shows good agreement with the seismic data in location and time evolution, with highest reflectivity in the south-central part of the network. On the contrary, the seismic data is poorly correlated with the ground rainfall accumulations derived from radar, which are very uniform all along the episode. This suggests that for this episode and zone, the estimations used to evaluate rainfall from radar data might be underestimated, probably due to beam blockage affecting low level precipitation.

**Figure 5:**
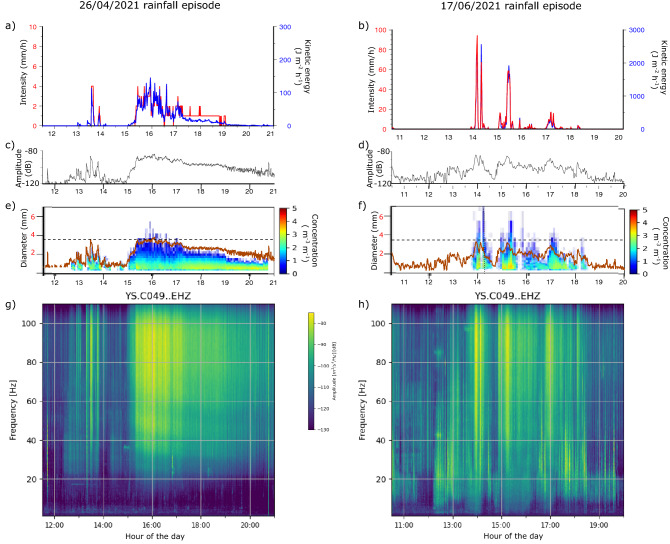
Disdrometer and seismic data at Das (central Cerdanya basin) for the April (**a**, **c**, **e**, **g**) and June (**b**, **d**, **f**, **h**) episodes. (**a**, **b**) Kinetic energy (red line) and rainfall intensity (blue line) obtained from disdrometer measurements. (**c**, **d**) Seismic amplitudes for frequencies between 40 and 120 Hz, expressed as dB. (**e**, **f**) Raindrop diameters and concentrations during each episode. Previously shown seismic amplitudes are repeated here (brown lines) for comparison with the raindrop information. Horizontal dashed lines mark the 3.5 mm diameter threshold level discussed in the text. (**g**, **h**) Spectrograms calculated using 120 s long windows.

The disdrometer observations available at Das, in the central zone of the seismic network, provide additional information on the rain intensity, its kinetic energy involved, and raindrop size distribution (Fig. 5a, c, e, g). After a first sub-event lasting 30 min and reaching peak intensities of 4.5 mm/h, the largest sub-event started around 15:00 and lasted for 6 h, with intensities reaching a peak value of 4.7 mm/h and totaling 12.8 mm of precipitation. Figure 5c show the amplitude of the seismic signal, filtered between 40 and 120 Hz, at station C049, located 380 m from the disdrometer. Figure 5e compares the raindrop diameters and concentration with this seismic signal. As expected, there is a consistent correlation with the rainfall intensity, recorded here with higher temporal resolution, the rain drop size and the seismic amplitudes at frequencies above 40 Hz (Fig. 5g). As discussed later, this seems related to the kinetic energy of raindrops transferred to the ground, a well-known fact in soil erosion studies^40,41^.

### June episode

The 7-day period presented in Fig. 3 (right panel) includes five main rainfall events, separated by periods of 1–2 days with small or null precipitation accumulations and well correlated with high values of seismic amplitude envelope. The first rainfall event (14 June) has a duration of just 1 h and corresponds to accumulated precipitations totaling 27.4 mm in the Puigcerdà rain gauge and is followed, 1 day later, by a smaller event. The main outburst, occurring the 17 June, largely exceeds these values, totaling 81.4 mm between 14:00 and 16:00 UTC in Puigcerdà. The last two events, occurring the 19 and 20 June, have longer time extent and correspond to rainfall accumulations of 9.1 and 14.3 mm at Puigcerdà. Note that the precipitation rates during the 14 and 17 June episodes largely overpass those in the April episode discussed previously.

**Figure 6:**
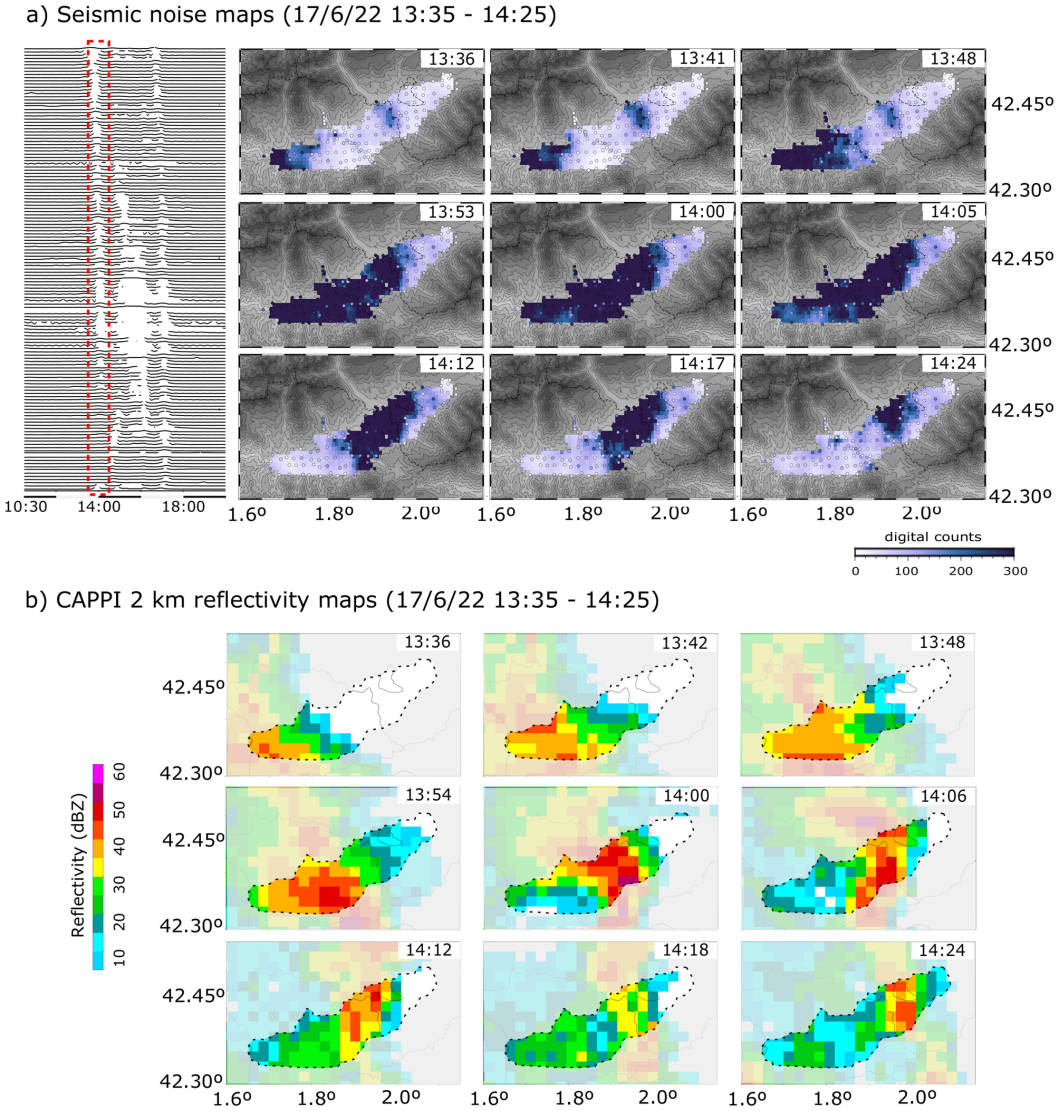
(**a**) Seismic amplitude maps during the 17 June 2021 between 13:35 and 14:25, shown in 6-min intervals. The inset shows the envelope of the seismic signal between 10:30 and 18:00 UTC, with white areas showing large amplitudes and the dashed box indicating the interval analyzed. (**b**) CAPPI reflectivity at 2 km height for the same intervals. The area covered by the seismic network is shown using grey mask. Topography is indicated using a grey color palette (see Fig. 1).

The animation provided as Supplementary Material 2 shows the evolution of the widespread 17/6/2022 rainfall episode at intervals of 15 min. In order to show the resolution power of the seismic data, we have analyzed a representative interval of this episode at intervals of 6 min and compared the results with the radar images. As for the April episode, the radar-derived maps for the full episode are provided as Supplementary Fig. 3. We have included in Fig. 6 the seismic and radar results for the first sub-event of this episode. Both the radar reflectivity and the seismic data clearly shows a rainfall band moving SW to NE. At 13:35 UTC the first indications of rainfall progression towards the east are observed at the westernmost seismic stations. About 20 min later, the affected area extends to longitudes of 1.8° and reaches Puigcerdà at about 13:55 UTC. During this time period, the radar reflectivity in the western part remains moderate, while the seismic amplitudes show high values (Suppl. Figure 3). As in the April case, this suggest that the radar derived precipitation estimations can be underestimated during this episode due to beam blockage and rainfall attenuation. Ground rainfall estimates derived from radar data appear to be rather uniform, in contrast with the large amplitude variations observed in seismic data. In this case, most of the areas with large radar reflectivity values are related to convective rainfall and correlated finely with the zones with largest seismic amplitudes.

Disdrometer and seismic data at Das are presented at Fig. 5b, d, f, h. Three sub-events can be identified, each lasting around 30 min. The peak intensities reach values of 94.6, 58.8 and 17.3 mm/h (Fig. 5b). Again, there is a convincing agreement between the seismic signal and the raindrop size distribution, with largest raindrops corresponding to peak seismic amplitudes (Fig. 5f). Regarding kinetic energy, the temporal correlation with seismic data is clear, but the large difference in peak energy between the April and June episodes (120 vs. 2500 J m^−2^ h^−1^) does not seem to affect the seismic amplitude, which shows similar values. This might indicate that above a threshold level about 3.5 mm, larger raindrops do not further increment the seismic signal amplitude.

## Seismic tracking of thunderstorms

Lightning strikes impulsively increases air temperatures along the stroke path to several thousand Kelvin degrees, resulting in a rapid, outward expansion of heated air as a shock wave that decays into a sonic pressure pulse, the thunder. This wave is produced at the same time that the lightning stroke and its location is distributed along the lightning path. While there are few examples of seismic records of the direct impact of lightning strikes^42^, the seismic recording of thunders is a well-known feature, resulting from the sonic-to-mechanical wave conversion near the recording point^43,44^. In some cases, it is also possible to identify the arrival of vibrations corresponding to sonic-mechanical conversions relatively far from the recording point that have then travelled as seismic surface waves. Although the electromagnetic sensor networks operated by meteorological agencies provide accurate lists of the lightning events over a region, they cannot provide information on the area where the associated thunders have been noted. As discussed below, seismic data can be useful to investigate the extension of this area, depending on factors as orography, humidity and temperature conditions or wind directions.

**Figure 7:**
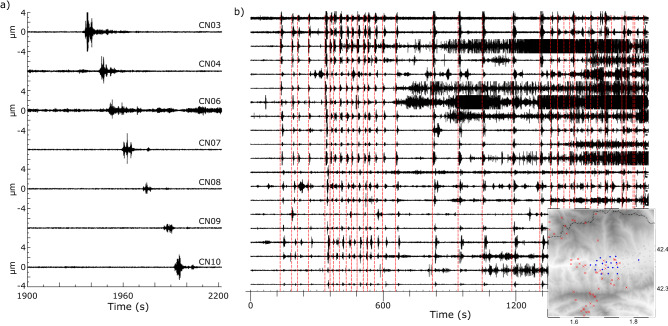
(**a**) Detection of signals related to a thunder sourced the 22 October 2019 using the broad-band seismic network. All the traces are shown at the same scale, with amplitudes expressed in μm/s. (**b**) 30 min of seismic data (15/6/2021 11:30–12:00 UTC, as recorded in the 20 eastern stations (blue dots in the inset map). Red dashed lines show the origin time of the lightning observations during the same time intervals, as reported by the SMC. Only the events located within less than 15 km of station C008 are included (plotted as red crosses in the inset map). Amplitudes are represented at a common scale and expressed in digital counts. Traces are ordered following longitude, with westward sites on top.

In most of the published contributions each thunder is recorded by only one or a few seismic stations. Figure 7a shows an example of such kind of results, corresponding to a thunder recorded during the 2019 broad-band deployment, including 10 broad-band stations distributed along the basin. The event was recorded over distances of around 25 km, from Bellver de Cerdanya to the west to Llo in the eastern Cerdanya (see Fig. 1 for location). The apparent velocity of the wave is about 335 m/s, consistent with an acoustic wave travelling in the atmosphere and coupling to ground vibration at the vicinity of each station.

Although this kind of result can provide relevant information on lighting generation and on the atmospheric conditions, the interest of recording thunders seismically is boosted when deployments of dense networks of recording stations are available, as shown by Zhu et al.^45^ , Hong et al.^46^ or Diaz et al.^47^. The data gathered in our experiment includes a large number of thunder signals recorded densely throughout the seismic network covering the Cerdanya basin. As an example, we present at Fig. 7b the seismic data during a 30-min interval immediately preceding the rainfall episode recorded the 15/6/2021 (see Fig. 3, right panel). The figure includes raw data recorded at the 20 stations located west of Bellver de Cerdanya. The large number of short-duration, high amplitude signals observed mostly in the first half of the period corresponds to thunder-generated signals, as evidenced by their propagation velocities of about 350 m/s and by their correlation with the lightning detections that are represented by red dashed lines in Fig. 7b. The figure inset shows the location of the recording stations and the lightning. Darker zones appearing to the end of the represented time interval correspond to the beginning of a rainfall episode similar to those described in the previous sections. As observed, when heavy rainfall does occur, the identification of thunder-related signals in the seismic data is no longer possible, as the vibrations induced by rain have higher amplitude than those due to the acoustic waves generated by thunders.

As discussed previously for rainfalls, seismic data can provide additional constraints on the evolution of thunderstorm. In the presented case, thundering signals are recorded 10–15 min before seismic signals related to rainfall. It is interesting to note that the occurrence of thunders during this storm seems to follow different temporal patterns that evolve during the episode. At the beginning of the episode, four thunders separated around 40 s are recorded, followed by a series of 11 seismic events that occur every 26 s. A few minutes later, 5 more thunders are recorded with regular spacing, in this case close to 2 min. Beside monitoring the temporal evolution of thundering, seismic data provide also a tool to range the sound intensity of each thunder; although the amplitude of the seismic signal recorded at each site depends on the distance to the origin, comparing the amplitudes of an individual thunder series recorded at multiple stations provides data to calibrate its relative seismic amplitudes. In this sense, the seismic records show larger amplitudes for the stations located to the SW, vanishing when moving due west. This locates the origin of the thunder near the SW of the network, consistently with the locations provided by meteorological data.

It should be possible to use the seismic recordings to locate the origin of thunders, but this would require knowing in some detail the temperature height profiles, as sound velocity is strongly affected by temperature. The short time interval between thunders and the relatively slow value of the sound velocity makes it difficult to identify the signals generated by the same event when the stations are far from the origin.

Details on the waveform and spectra of thunder-related signals are provided in Suppl. Figure 4. Large differences do occur between stations recording a single thunder, due to the distance to the origin, but also to topography effects (reverberations), different coupling degree, etc. The signals tend to have a sharp onset and a complex time/amplitude evolution, clearly different from what is observed for earthquakes or quarry blast explosions. Similar to the waveforms wiggles, the frequency content between the different stations recording a single event differs, as illustrated in Suppl. Figure 4, showing a station dominated by frequencies between 70 and 120 Hz, a site with a rather homogeneous frequency content ranging between 10 and 100 Hz and a third site with maximum energy in the 20–50 Hz band.

**Figure 8:**
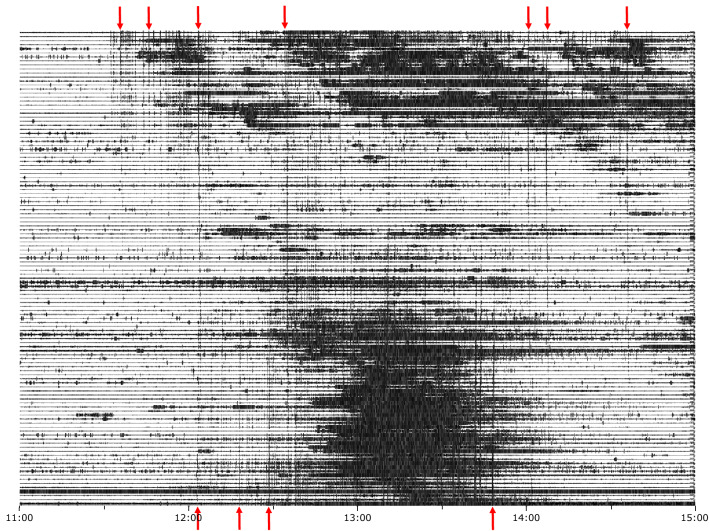
Four hours of seismic data recorded the 15 June 2021 (11:00–15:00 UTC) across the high-density seismic network, showing the signals generated by rainfall and thunders. The seismic data has been high-pass filtered above 30 Hz. The stations are ordered accordingly to their longitude, with the westernmost stations on top. Red arrows show some of the thunder-related signals. Red arrows highlight some of the thunder-related events.

To discuss the relationship between the signals related to rainfall and thunders, Fig. 8 shows 4 h of seismic data recorded the 15 June 2021 (11:00–15:00 UTC) across the high-density seismic network. The thunder-related signals appear as almost vertical lines covering total or partially the station network, while the blackish intervals shown the occurrence of heavy rain; the first thunder-related signals at the western stations (top) are those discussed in the previous figure. Figure 3 (right panel) also shows that rainfall during this episode is more intense and more concentrated in time at the stations located to the east (bottom of the figure). The western stations show more sparse rainfall, extending during most of the represented time interval. The stations located in the center of the basin show no evidence of rainfall.

The analysis of the continuity of the thunder-related events throughout the network shows that there are at least two or three thunderstorm convective cores. Firstly, thunders are only recorded to the west and propagate west to east, hence pointing to a thunderstorm located SW of the network. Around noon the thundering activity in the western part of the investigated area disappears, shifting to the central and eastern part of the network, with thunder signals propagating west to east. Between 12:45 and 13:45 UTC, the large seismic amplitudes generated by rainfall hides the detection of thunder-related signals in the eastern part of the network. Around 14:30 UTC data show thundering activity in the west, with events detections persisting through the mid-eastern part of the network. Some of the events are recorded throughout the whole network, that spans 40 km between its eastern and western limits (300 km^2^).

## Discussion and conclusions

Our contribution to environmental seismology shows that high density seismic network deployments can be used to analyze the detailed evolution over space and time of meteorological events such as rainfall episodes or thunderstorms. Data acquired using a seismic network covering the Cerdanya basin in the eastern Pyrenees, with an interstation distance of 1.5 km, has provided strong evidence that high frequency seismic noise for frequencies above 40 Hz can be a proxy for rainfall.

Comparing seismic spectra and rainfall measures in meteorological stations, we have observed an excellent correlation between rainfall episodes and high seismic amplitudes for frequencies above 40 Hz. Other sources of vibration, mostly related to anthropogenic activities, can contribute to the seismic amplitude in this band. However, the consistency between the seismic record on a relevant number of neighboring stations, altogether with their correlation with rainfall measurements at the reference meteorological stations, strongly suggests that the dominant source of the noise in this band is rainfall, as anthropogenic signals are known to attenuate over distances of few hundred of meters. Using many stations has allowed us to identify and discard a limited number of sites (below 6%) with strong signals related to local sources such as human activities.

Rainfall has been observed in multiple seismic studies, many of them related to the so-called “fluvial seismology”^48,49^. However, few contributions analyze detailed the relationships between rainfall physical properties, as the drop size, and seismic amplitudes. Dean^50^ concluded that seismic signals related to the impact of rain drops on the ground have frequencies above 80 Hz and can be detected at offsets up to 0.8 m. Recently, Bakker et al.^51^ have modeled the relation between seismic power at frequencies above 50 Hz and rainfall intensity and kinetic energy estimations derived from disdrometer data, showing that the relationships are exponential and that up to 90% of the seismic power is related to the small fraction of drops larger than 3 mm. The authors conclude that seismic data can be particularly useful in soil erosion studies.

Our study details two rainfall events of different characteristics that quantifies how seismic amplitudes can be used to constrain the spatial and temporal localization of large precipitation episodes. The first one, recorded the 26 April 2021, correspond to a rainfall traveling west to east along the Cerdanya basin with an apparent speed, derived from the analysis of the seismic data, of 4.5 m/s. Disdrometer data at Das, near the center of our network, shows the first sub-event, with a duration of around 30 min and peak intensities of 4.5 mm/h, followed by a longer episode, lasting for 6 h, with intensities reaching a peak value of 4.7 mm/h and totaling 12.8 mm of precipitation. Meteorological radar data shows that the rainfall during this episode is of stratified type. The second episode, recorded the 17 June 2021, accumulated 31.5 mm at Das between 13:30 and 18:00, distributed in three sub-events, each lasting around 30 min. The intensity values recorded are much higher than in the April episode, reaching values now close to 100 mm/h; radar data indicate this rain type is convective.

Seismic amplitude variations follow a similar evolution when compared to the 2 km height CAPPI radar reflectivity data but they differ from the ground rainfall estimates derived from radar data in some periods. Weather radar precipitation estimates provides a spatial resolution of the order of one square km, but are subjected to a conversion process to pass from the measured reflectivity to rainfall estimations. This conversion is usually more uncertain in complex orography due to effects such as radar beam blockage, attenuation by heavy rainfall or low-level circulations induced by the terrain. This suggests that for this area with complex topographic relief, the rainfall maps derived from radar are less accurate, and that seismic data recorded on a dense network may better estimate of ground surface rainfall intensity. The high spatial resolution of our network, altogether with the fine time sampling used in seismological studies, makes possible to resolve details of storm time and spatial evolution that cannot be achieved otherwise.

Seismic power values during both rain events are similar, and peak around − 85 dB (Fig. 5). This is consistent with the exponential relationship between seismic PSD and rainfall intensity pointed out by Bakker et al.^51^. While raindrops have diameters below 3.5 mm, the seismic amplitudes increase with raindrop size. However, during the few periods that rain drops diameters exceeded this value, seismic amplitudes did not increase, suggesting that there is a threshold value from which seismic data are no longer proportional to the size of the raindrop impacts. This will also explain the similar amplitude values during the April and June episodes, in despite of the large differences in kinetic energy.

The amplitude at some of the stations located near the Carol River, seems to reflect the mixed effect of rainfall and increased river discharge due to previous upstream rainfall. This would explain why those stations remain with large amplitude levels once rainfall has ended in their region. However, the stations located close to the Segre River, the main river in the region, does not show this behavior. We speculate that this difference can be related to the fact that the rainfall episodes moved west to east; as the Segre River catchment is located eastward of the area covered by the seismic network, the effects of local rainfall and discharge in the upper catchment are not synchronous in the Cerdanya basin.

Regarding thunderstorms, our contribution proves that the sonic waves generated by thunders can be recorded seismically at distances reaching few tens of kilometers. Seismic recording of thunders allows to infer not only the rain fall episodes occurring with lightning, but also the strength of the associated shock wave, opening the door to build “intensity maps” like those currently produced for seismic events. However, periods with heavy rainfall can mask the detection of thunder-generated signals. Meteorological lightning detection networks provide a catalogue of the lightning flashes, including their location, origin time and, in the case of lightning reaching the ground, their current intensity. The seismic data can potentially be used to locate the origin of each thunder by raytracing along the air-wave propagation path. However, it is well known that the number of lightning flashes during a single storm can reach very high numbers and that the events can be very close in time. This can be problematic for locating events using seismic data, as it will be difficult to associate properly the detections at each station with the correspondent event. The sonic wave generated by thunders is expected to be affected by factors as temperature, air humidity, and wind direction. As the location of the lightning generating each thunder can be obtained from electromagnetic sensors, it should be possible to design an inversion procedure of the seismic data to investigate these atmospheric properties variations during thunderstorms.

In summary, our contribution shows that elevated seismic noise at frequencies above 40 Hz can be used to identify the arrival of rainfall. The origin of these vibrations is related to the impact of rain drops, with increasing drop size resulting in increased seismic amplitudes for drop diameters below 3.5 mm. Beyond this value, seismic data seem to saturate and are no longer proportional to the size of the raindrop impacts. We have also shown that, in some specific cases, seismic data can be a more accurate diagnostic of rainfall than the precipitation estimations derived from radar data. Finally, we prove that thunder-related acoustic signals can be detected seismically at distances of few tens of kilometres, providing a new tool to investigate the atmospheric properties during storms. We are convinced that this contribution proves that seismic data can provide significant information for meteorological studies, particularly in relation to the temporal and spatial evolution of rainfall events in mountain areas. Since numerous seismic surveys that use dense networks record background seismic noise, we suggest that meteorologists can collaborate to produced multi-disciplinary datasets that quantify storm activity.

## Methods

Seismic waveforms and record section plots are produced using the ObsPy software^52,53^ and the Seismic Analysis Code (SAC)^54^.

Maps are generated using the Generic Mapping Tools (GMT) code^38^ and the topographic database is retrieved from CIAT-CSI SRTM (http://srtm.csi.cgiar.org)^24^.

Spectrograms are obtained by dividing the signal in overlapping intervals of a given length and calculating the Power Spectral density in each of them, using the ObsPy software ^52,53^. The spectrograms in Fig. 2 are calculated using a window length of 1800s and an overlap of 50%, while those in Fig. 5 are calculated with a window length of 120 s and the same overlap rate. In both cases, a color palette, expressed in dB and relative to a reference value of 1 (m^2^/s^4^)/Hz, is used to show the energy distribution.

The grided maps presented in Figs. 4 and 6, as well as the animations included as Supplementary Materials 1 and 2 have been constructed using the nearest neighbor algorithm included in the GMT^38^ package, using a search radius of 2 min of arc, an increment of 0.4 min of arc and requiring that 5 of 6 azimuthal sectors with data for averaging.

## Supplementary Information

Supplementary Video 1.

Supplementary Video 2.

Supplementary Figures.

## Data Availability

The seismic dataset will be publicly distributed through the Inst. Cartogràfic I Geològic de Catalunya (ICGC) EIDA Data Center (http://ws.icgc.cat/fdsnws) at the end of the embargo period. In the meanwhile, data is available upon request at the Geo3Bcn-CSIC data center (http://eida.geo3bcn.csic.es:8080/fdsnws/). Data from the permanent meteorological network managed by the Meteorological Service of Catalonia (SMC) is available through its data portal: https://analisi.transparenciacatalunya.cat/Medi-Ambient/Dades-meteorol-giques-de-la-XEMA/nzvn-apee.

## References

[1] Díaz J (2016). On the origin of the signals observed across the seismic spectrum. Earth Sci. Rev..

[2] Campillo M, Paul A (2003). Long range correlations in the diffuse seismic coda. Science.

[3] Larose E (2015). Environmental seismology: What can we learn on earth surface processes with ambient noise?. J. Appl. Geophys..

[4] Gutenberg B (1958). Microseism. Adv. Geophys..

[5] Grob M, Maggi A, Stutzmann E (2011). Observations of the seasonality of the Antarctic microseismic signal, and its association to sea ice variability. Geophys. Res. Lett..

[6] Podolskiy EA, Walter F (2016). Cryoseismology. Rev. Geophys..

[7] Burtin A, Bollinger L, Vergne J, Cattin R, Nábělek JL (2008). Spectral analysis of seismic noise induced by rivers: a new tool to monitor spatiotemporal changes in stream hydrodynamics. J. Geophys. Res. Solid Earth.

[8] Díaz J, Sánchez-Pastor P, Ruiz M (2019). Hierarchical classification of snowmelt episodes in the Pyrenees using seismic data. PLoS One.

[9] Clements T, Denolle MA (2018). Tracking groundwater levels using the ambient seismic field. Geophys. Res. Lett..

[10] Rindraharisaona E (2022). Seismic signature of rain and wind inferred from seismic data. Earth Planets Space.

[11] Withers MM, Aster RC, Young CJ, Chael EP (1996). High-frequency analysis of seismic background noise as a function of wind speed and shallow depth. Bull. Seismol. Soc. Am..

[12] Tape C, Ringler AT, Hampton DL (2020). Recording the Aurora at Seismometers across Alaska. Seismol. Res. Lett..

[13] Díaz J, Ruiz M, Curto JJ, Torta JM, Ledo J, Marcuello A, Queralt P (2020). On the observation of magnetic events on broad-band seismometers. Earth Planets Space.

[14] de Groot-Hedlin CD, Hedlin MAH (2014). Infrasound detection of the Chelyabinsk meteor at the USArray. Earth Planet. Sci. Lett..

[15] Gaspà Rebull O, Cusí JD, Ruiz Fernández M, Muset JG (2006). Tracking fin whale calls offshore the Galicia Margin, North East Atlantic Ocean. J. Acoust. Soc. Am..

[16] O’Connell-Rodwell CE (2007). Keeping an “ear” to the ground: seismic communication in elephants. Physiology.

[17] Mortimer B, Rees WL, Koelemeijer P, Nissen-Meyer T (2018). Classifying elephant behaviour through seismic vibrations. Curr. Biol..

[18] Gabàs A (2016). Joint audio-magnetotelluric and passive seismic imaging of the cerdanya basin. Surv. Geophys..

[19] Conangla L (2018). Cold-air pool evolution in a wide Pyrenean valley. Int. J. Climatol..

[20] Miró JR, Peña JC, Pepin N, Sairouni A, Aran M (2018). Key features of cold-air pool episodes in the northeast of the Iberian Peninsula (Cerdanya, eastern Pyrenees). Int. J. Climatol..

[21] Udina M (2020). Multi-sensor observations of an elevated rotor during a mountain wave event in the Eastern Pyrenees. Atmos. Res..

[22] Gonzalez S (2019). Decoupling between precipitation processes and mountain wave induced circulations observed with a vertically pointing k-band doppler radar. Remote Sens. (Basel).

[23] Trapero L, Bech J, Duffourg F, Esteban P, Lorente J (2013). Mesoscale numerical analysis of the historical November 1982 heavy precipitation event over Andorra (Eastern Pyrenees). Nat. Hazard..

[24] Jarvis, A., Reuter, H. I., Nelson, A. & Guevara, E. Hole-filled seamless SRTM data V4, in *International Centre for Tropical Agriculture (CIAT)*. https://srtm.csi.cgiar.org (2008).

[25] Meng H, Ben-Zion Y, Johnson CW (2021). Analysis of seismic signals generated by vehicle traffic with application to derivation of subsurface Q-values. Seismol. Res. Lett..

[26] Llabrés-Brustenga A, Rius A, Rodríguez-Solà R, Casas-Castillo MC, Redaño A (2019). Quality control process of the daily rainfall series available in Catalonia from 1855 to the present. Theor. Appl. Climatol..

[27] Casellas E (2020). A meteorological analysis interpolation scheme for high spatial-temporal resolution in complex terrain. Atmos. Res..

[28] Altube P, Bech J, Argemí O, Rigo T (2015). Quality control of antenna alignment and receiver calibration using the sun: adaptation to midrange weather radar observations at low elevation angles. J. Atmos. Ocean Technol..

[29] Bech J, Codina B, Lorente J, Bebbington D (2003). The sensitivity of single polarization weather radar beam blockage correction to variability in the vertical refractivity gradient. J. Atmos. Ocean Technol..

[30] Trapero L, Bech J, Rigo T, Pineda N, Forcadell D (2009). Uncertainty of precipitation estimates in convective events by the meteorological service of Catalonia radar network. Atmos. Res..

[31] Villarini G, Krajewski WF (2010). Review of the different sources of uncertainty in single polarization radar-based estimates of rainfall. Surv. Geophys..

[32] Powell SW, Houze RA, Brodzik SR (2016). Rainfall-type categorization of radar echoes using polar coordinate reflectivity data. J. Atmos. Ocean Technol..

[33] Pineda N, Montanyà J, Betz HD, Schumann U, Laroche P (2008). Lightning detection in Spain: the particular case of catalonia. Lightning: Principles, Instruments and Applications.

[34] Löffler-Mang M, Joss J (2000). An optical disdrometer for measuring size and velocity of hydrometeors. J. Atmos. Ocean Technol..

[35] Tokay A, Wolff DB, Petersen WA (2014). Evaluation of the new version of the laser-optical disdrometer, OTT parsivel2. J. Atmos. Ocean Technol..

[36] de Angelis S, Bodin P (2012). Watching the Wind: Seismic data contamination at long periods due to atmospheric pressure-field-induced tilting. Bull. Seismol. Soc. Am..

[37] Smith K, Tape C (2019). Seismic noise in central Alaska and influences from rivers, wind, and sedimentary basins. J. Geophys. Res. Solid Earth.

[38] Wessel P, Smith WHF, Scharroo R, Luis J, Wobb F (2013). Generic mapping tools. Eos (Washington DC).

[39] Soler A, Pineda N, San Segundo H, Bech J, Montanyà J (2021). Characterisation of thunderstorms that caused lightning-ignited wildfires. Int. J. Wildland Fire.

[40] Serio MA, Carollo FG, Ferro V (2019). Raindrop size distribution and terminal velocity for rainfall erosivity studies. A review. J. Hydrol. (Amst).

[41] Cerro C, Bech J, Codina B, Lorente J (1998). Modeling rain erosivity using disdrometric techniques. Soil Sci. Soc. Am. J..

[42] Hinzen KG (2012). Seismological analysis of a lightning strike. Seismol. Res. Lett..

[43] Kappus ME, Vernon FL (1991). Acoustic signature of thunder from seismic records. J. Geophys. Res..

[44] Lin TL, Langston CA (2007). Infrasound from thunder: a natural seismic source. Geophys. Res. Lett..

[45] Zhu T, Stensrud DJ (2019). Characterizing thunder-induced ground motions using fiber-optic distributed acoustic sensing array. J. Geophys. Res. Atmos..

[46] Hong T-K, Park S, Chung D, Kim B (2022). Inversion of acoustic thunder source spectral model from thunder-induced seismic waves in megacity. Geophys. J. Int..

[47] Díaz J, DeFelipe I, Ruiz M, Andrés J, Ayarza P, Carbonell R (2022). Identification of natural and anthropogenic signals in controlled source seismic experiments. Sci. Rep..

[48] Burtin A (2011). Towards the hydrologic and bed load monitoring from high-frequency seismic noise in a braided river: the ‘torrent de St Pierre’ French Alps. J. Hydrol. (Amst).

[49] Roth DL (2016). Bed load sediment transport inferred from seismic signals near a river. J. Geophys. Res. Earth Surf..

[50] Dean T (2017). The seismic signature of rain. Geophysics.

[51] Bakker M (2022). Seismic modelling and observations of rainfall. J. Hydrol. (Amst).

[52] Krischer L (2015). ObsPy: a bridge for seismology into the scientific python ecosystem. Comput. Sci. Discov..

[53] Megies T, Beyreuther M, Barsch R, Krischer L, Wassermann J (2011). ObsPy—What can it do for data centers and observatories?. Ann. Geophys..

[54] Goldstein P, Dodge D, Firpo M, Lee M, Lee W, Knamori H, Jennings P, Kisslinger C (2003). SAC2000: signal processing and analysis tools for seismologists and engineers. The IASPEI International Handbook of Earthquake and Engineering Seismology.

