# Supplementary material for: Monitoring storm evolution using a high-density seismic network
Source: Sci Rep. 2023 Feb 1;13:1853. doi: 10.1038/s41598-023-28902-8 (PMC9892581; doi:10.1038/s41598-023-28902-8)
Supplement: Supplementary file 3 — Supplementary Figures. [file 41598_2023_28902_MOESM3_ESM.docx]

**Supplementary Material**

**Supplementary Material 1:** Animation of the seismic amplitude variations during the April 2021 rainfall episode, starting the 25 Abril at 00:00 and ending the 1^st^ May at 23:45. Each snapshot correspond to an interval of 15 minutes.

**Supplementary Material 2:** Animation of the seismic amplitude variations during the June 2021 rainfall episode, starting the 14 June at 00:00 and ending the 20 June at 23:45. Each snapshot correspond to an interval of 15 minutes.


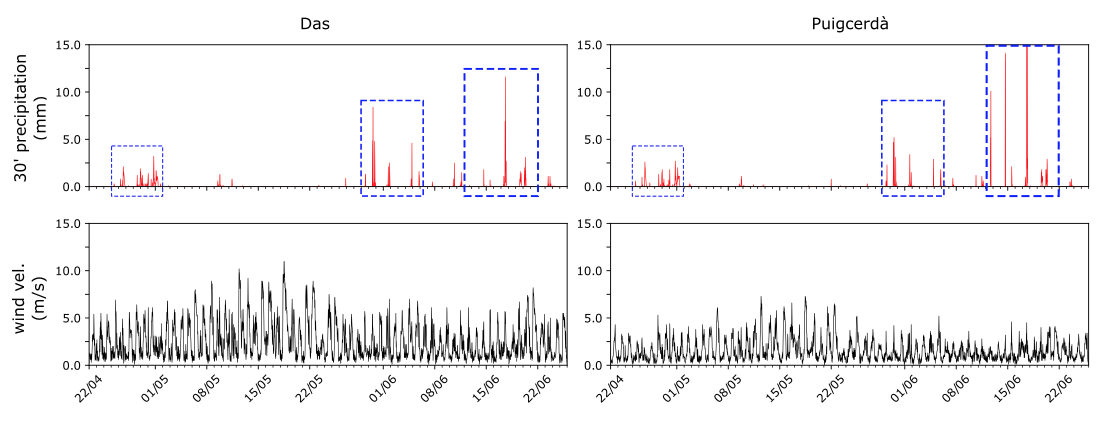


**Supplementary Figure 1:** *Precipitation and wind speed data for the meteorological stations at Das and Puigcerdà, Blue dashed boxes show the investigated rainfall episodes*

*
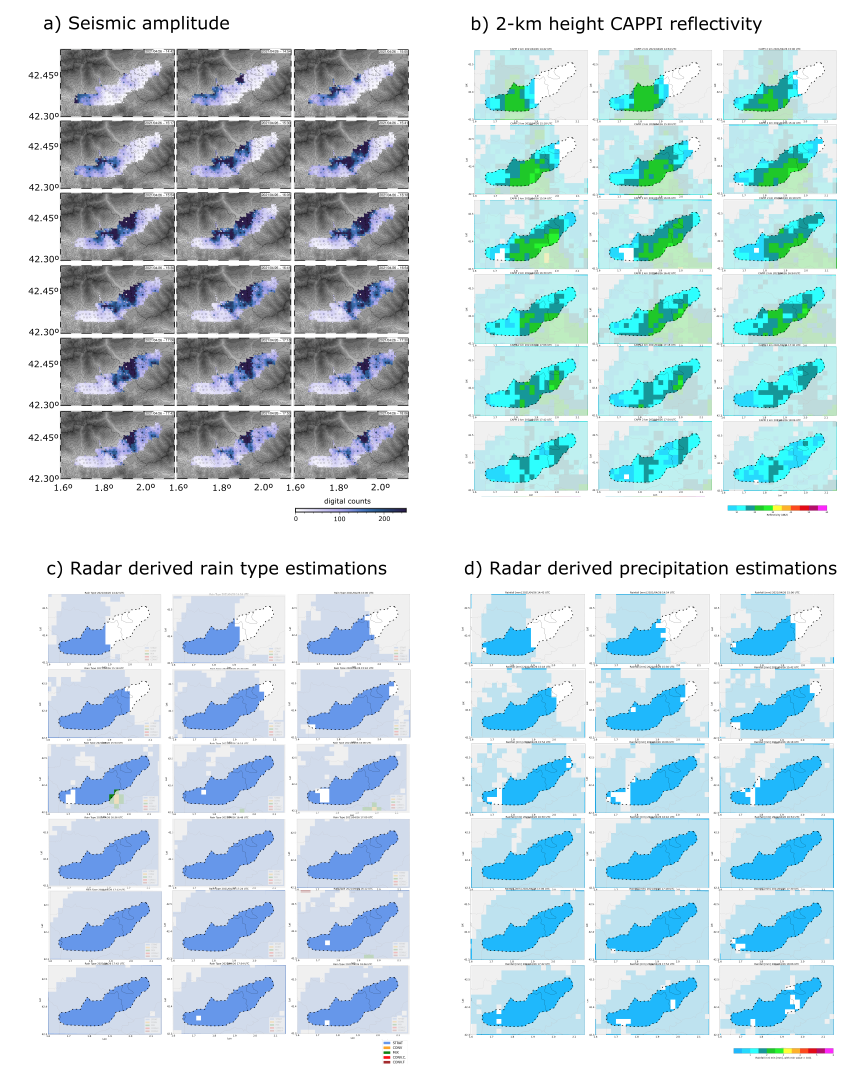
*

**Supplementary Figure 2:** Comparison between seismic amplitudes and meteorological radar products for the April 2021 event. a) seismic amplitude; b) 2-km height CAPPI reflectivity; c) radar-derived rain type estimations; d) radar-derived precipitation estimations. Snapshots each 12 minutes, starting the 26 April 2021 at 14:42 and ending 26 April 2021 at 18:06.

**
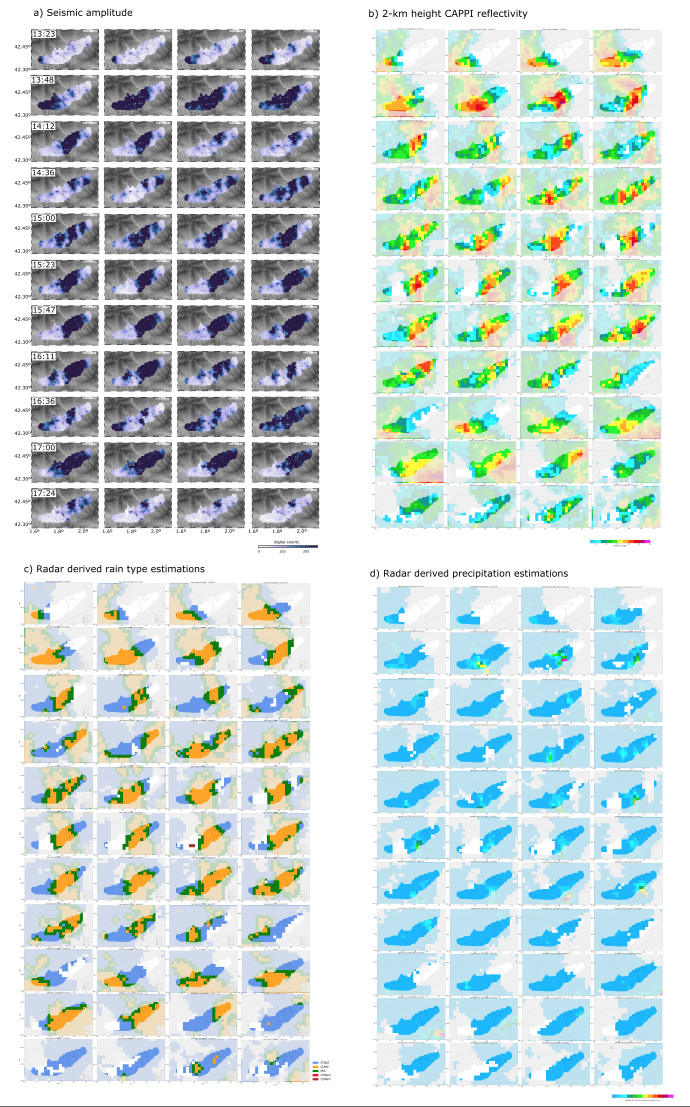
**

**Supplementary Figure 3:** Comparison between seismic amplitudes and meteorological radar products for the June 2021 event. a) seismic amplitude; b) 2-km height CAPPI reflectivity; c) radar-derived rain type estimations; d) radar-derived precipitation estimations. Snapshots each 12 minutes, starting the 17 June 2021 at 13:24 and ending the 17 June 2021 17:42.


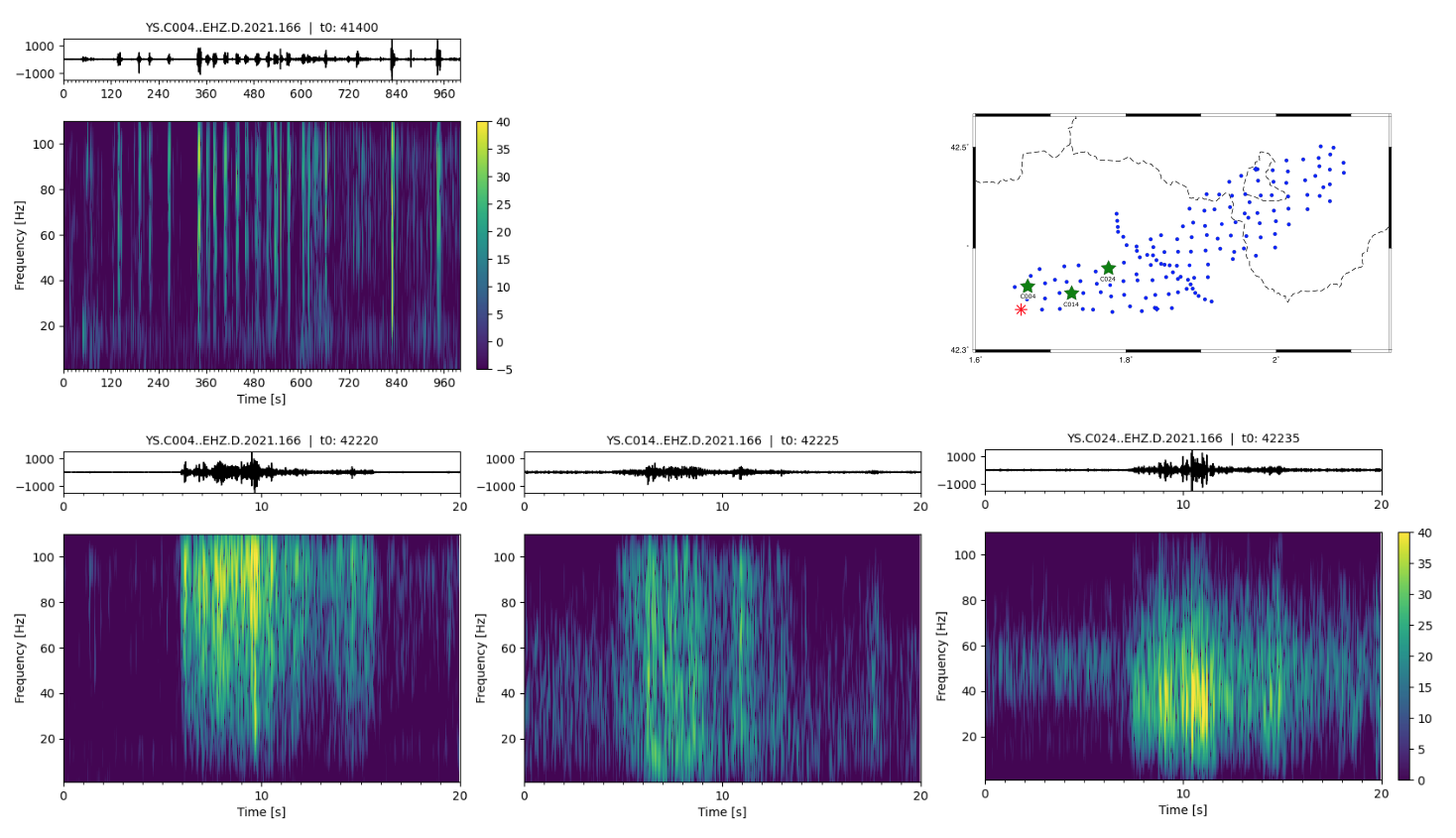


**Supplementary Figure 4:** Spectrograms of a thunder-related event the 15 June 2021 recorded at stations C004, C014 and C024, all located at the western part of the array. The upper panel shows 17 min of data including a thunder series as recorded at station C004. Lower panel zoom in an individual event as recorded at the three selected stations. The map inset show the location of these stations (green stars) and the lightning location provided by the Meteorological Service of Catalonia (red star).
